# Ventilatory Complexity Persists in *Phox2b* Mutant Mice Lacking the Retrotrapezoid Nucleus/Parafacial Respiratory Group (RTN/pFRG) and in Humans With Congenital Central Hypoventilation Syndrome

**DOI:** 10.1002/cne.70117

**Published:** 2025-12-03

**Authors:** Christian Straus, Anja Ranohavimparany, Nelina Ramanantsoa, Lysandre Tremoureux, Maxime Patout, Marie‐Noëlle Fiamma, Florence Cayetanot, Boris Matrot, Jorge Gallego, Laurence Bodineau, Thomas Similowski

**Affiliations:** ^1^ Sorbonne Université, INSERM, UMRS1158 Neurophysiologie Respiratoire Expérimentale et Clinique Paris France; ^2^ AP‐HP, Groupe Hospitalier Universitaire APHP‐Sorbonne Université, hôpital Pitié‐Salpêtrière, Service des Explorations Fonctionnelles de la Respiration, de l'Exercice et de la Dyspnée, Département R3S Paris France; ^3^ Université Paris Cité, Inserm UMR 1141, NeuroDiderot Paris France; ^4^ AP‐HP, Groupe Hospitalier Universitaire APHP‐Sorbonne Université, hôpital Pitié‐Salpêtrière, Service des Pathologies du Sommeil & Centre de Référence Maladies Rares “Hypoventilation Centrales Congénitales”, Département R3S Paris France; ^5^ AP‐HP, Groupe Hospitalier Universitaire APHP‐Sorbonne Université, hôpital Pitié‐Salpêtrière, Département R3S Paris France

**Keywords:** central respiratory pattern generators, congenital central alveolar hypoventilation, control of breathing, mathematical complexity, *Phox2b* mutations, variability

## Abstract

Breathing is inherently variable due to the nonlinear dynamics of its brainstem neural control. In amphibians, a gill and a lung oscillator interact to produce breathing but the lung oscillator is necessary and sufficient to produce a mathematically complex behavior. In mammals, where the preBötzinger complex (preBötC) is considered homologous to the amphibian lung oscillator and the retrotrapezoid nucleus/parafacial respiratory group (RTN/pFRG) homologous to the amphibian gill oscillator, the origin of ventilatory complexity is not known. We address this question by characterizing ventilation variability in *Phox2b* mutant mice lacking the RTN/pFRG and human patients with *Phox2b* mutation‐confirmed congenital central hypoventilation syndrome (CCHS). Ventilatory recordings were obtained from *Phox2b* ^27^ *^ala/+^*—dying within hours after birth—and *Egr2^cre/+^*; *Phox2b^27ala/+^* mice—generally surviving until adulthood—and their wild‐type (WT) littermates, during behavioral quiescence at various developmental stages. Human data were collected from CCHS patients and healthy controls during quiet wakefulness. Variability was assessed using the coefficient of variation, complexity using noise titration (noise limit, NL), and sensitivity to initial conditions using the largest Lyapunov exponent (LLE). Mice from both mutant lineages exhibited greater variability at early developmental stages, which decreased with maturation in *Egr2^cre^* ^/+^; *Phox2b^27ala/+^* mice. NL was consistently higher in mutant mice than in WT, indicating preserved or even enhanced ventilatory complexity despite RTN/pFRG dysfunction. CO_2_ reduced variability but did not affect complexity. In humans, no differences were observed between patients and controls for variability, NL, or LLE. Ventilatory complexity persists in mice lacking a functional RTN/pFRG and despite *Phox2b* mutations in humans, suggesting that the pontomedullary rhythm and pattern generators that include the preBötC may be its principal source. This supports the analogy between mammalian and amphibian rhythm generators.

## Graphical abstract

*Phox2B* mutant mice and patients with congenital central hypoventilation syndrome exhibit ventilatory variability and complexity comparable to those of controls, suggesting that the RTN/pFRG nucleus is not required for brainstem central pattern generators to generate complex breathing.

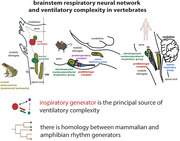

## 1 Introduction

**FIGURE 1:**
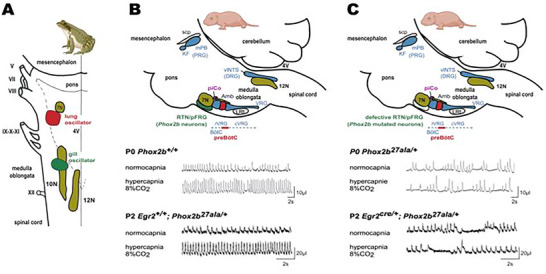
**Respiratory central pattern generators amphibian–mammalian analogy**. (A) Putative localization of gill (green) and lung (red) oscillators in amphibian on a dorsal view of the brainstem. (B) Location of the three respiratory rhythm oscillators identified in mice within the pontomedullary respiratory neural network on a sagittal view of the brainstem (preBötC, red; pFRG tightly bound to the RTN, green; piCo, purple). Tracings illustrate the ventilation of wild‐type mice under normocapnia and hypercapnia (Ramanantsoa et al. 2011). (C) Schematic representation of the respiratory neural network that highlights the recognized impairment of the RTN/pFRG induced by the *Phox2b* gene mutation. Tracings below illustrate the ventilation in normocapnia and hypercapnia of *Phox2b* mutant mice (constitutive mutation, *Phox2b^27ala/+^* and restricted RTN/pFRG mutation, *Egr2^cre^* ^/^ *^+^*; *Phox2b^27ala/+^* (Ramanantsoa et al. 2011). 10N, dorsal motor of the vagus nucleus; 12N, hypoglossal nucleus; 7N, facial nucleus; Am, ambiguus nucleus; BötC, Bötzinger complex; cVRG, caudal ventral respiratory group; DRG, dorsal respiratory group; IX, glossopharyngeal nerve; KF, Kolliker–Füse; LRt, lateral reticular nucleus; mBP, median part of the parabrachial nucleus; piCo, post‐inspiratory complex; preBötC, pre‐Bötzinger complex; PRG, pontin respiratory group; RTN/pFRG, retrotrapezoid nucleus/parafacial respiratory group; rVRG, rostral ventral respiratory group; scp, superior cerebellar peduncle; V, trigeminal nerve; VII, facial nerve; VII, vestibulocochlear nerve; vlNTS, ventrolateral subdivision of the nucleus of the solitary tract; VRG, ventral respiratory group; X, vagal nerve; XI, accessory nerve; XII, hypoglossal nerve. *Source:* (A) License number 6015280056533 for the adapted version of frog oscillator localization (Vasilakos et al. 2005). (B) Copyright 2008, National Academy of Sciences, USA for the *Phox2b^+/+^* and *Phox2b^27ala/+^* tracings adapted from Dubreuil et al. (2008) (https://creativecommons.org/licenses/by‐nc‐sa/3.0/ for the *Egr2^+/+^*; *Phox2B^27ala/+^* and *Egr2^cre/+^*; *Phox2B^27ala/+^* tracings) (Ramanantsoa et al. 2011). (C) Copyright 2008, National Academy of Sciences, USA for the *Phox2b^+/+^* and *Phox2b^27ala/+^* tracings adapted from Dubreuil et al. (2008) (https://creativecommons.org/licenses/by‐nc‐sa/3.0/ for the *Egr2^+/+^*; *Phox2B^27ala/+^* and *Egr2^cre/+^*; *Phox2B^27ala/+^* tracings) (Ramanantsoa et al. 2011). Frog drawing (A) is adapted from “Wood frog (Rana sylvatica)” and newborn mice drawing (B) is adapted from “Mouse pup (young)”, both by BioRender.com 2025, retrieved from https://app.biorender.com/biorender‐templates.

Breathing in vertebrates is inherently cyclical but not strictly regular. This irregularity is particularly evident in discontinuous patterns, such as episodic breathing, but is also present in continuous breathing, as demonstrated by breath‐by‐breath variability in amplitude and timing. Such variability arises from the mathematically complex nature of the brainstem‐generated neural drive to breathe (Hess et al. 2013; Straus et al. 2011). The source of this complexity has been elucidated in amphibians, where the neural drive to breathe arises from two central pattern generators: one governing gill ventilation and the other governing lung ventilation. Isolated tadpole brainstems produce complex fictive breathing that can be analyzed using various metrics, such as noise titration and other mathematical indicators (Straus et al. 2011). Previous studies have shown that the lung oscillator is both necessary and sufficient to generate the complexity of the fictive ventilatory behavior observed in vitro in the isolated brainstem of post‐metamorphic tadpoles (Ranohavimparany et al. 2016). Specifically, inhibiting the lung oscillator using DAMGO significantly reduced or abolished the complexity of the brainstem's global neural output (Ranohavimparany et al. 2016), whereas inhibiting the gill oscillator by altering chloride concentrations had no such effect (Ranohavimparany et al. 2016). In mammals, the neural drive to breathe also originates from several central pattern generators (Anderson and Ramirez 2017), two of which—the preBötzinger complex (preBötC) and the parafacial respiratory group (pFRG)—are analogs and proposed homologs of the amphibian lung and gill oscillators, respectively (Figure 1) (Vasilakos et al. 2005; Wilson et al. 2006). However, their respective contributions to ventilatory complexity remain unknown.

The *Phox2b* gene, located on chromosome 4 in humans, encodes a homeodomain transcription factor expressed in the nervous system. This factor plays a critical role in the development of neural crest derivatives and the regulation of visceral innervation. In humans, heterozygous mutations in this gene—most commonly an expansion of 5–10 alanine residues within a series of 20 residues (Amiel et al. 2003)—result in a complex dysautonomia. It is generally termed congenital central hypoventilation syndrome (CCHS) because sleep‐related hypoventilation and lack of carbon dioxide sensitivity dominate the prognosis, but it also involves cardiac conduction abnormalities, impaired development of enteric ganglia responsible for Hirschsprung's disease, sympathetic tumors, and other abnormalities occurring in various combinations.

Mice carrying a constitutive mutation in the *Phox2b* gene identical to one found in patients with congenital central alveolar hypoventilation—a 7‐alanine expansion—exhibit a developmental defect in the retrotrapezoid nucleus (RTN), a brainstem structure overlapping with the pFRG (Dubreuil et al. 2008). Although these mice (*Phox2b^27ala/+^*) are not viable beyond the neonatal period, they survive long enough after birth to allow for respiratory recordings. A complementary model of retrotrapezoid nucleus/parafacial respiratory group (RTN/pFRG) dysfunction is provided by knock‐in mice in which the *Phox2b* mutation is conditional and restricted to RTN/pFRG neurons (Ramanantsoa et al. 2011). These conditional mutant mice (*Egr2^cre^* ^/^ *^+^*; *Phox2B^27ala/+^*) display pronounced hypoventilation and a complete lack of carbon dioxide sensitivity at birth (as their *Phox2b^2ala/+^* counterparts). However, most of these animals survive to adulthood, during which they exhibit normalized ventilation and partial restoration of chemosensitivity (Ramanantsoa et al. 2011). Both of the above genetically modified populations serve as models of “breathing without the RTN/pFRG” (or at best with a defective RTN/pFRG).

We hypothesized that analyzing the breathing patterns of these mice would provide insights into the origins of ventilatory complexity in mammals. Within the framework of the gill–lung and preBötC‐RTN/pFRG homology hypothesis, we further hypothesized that ventilatory complexity would be comparable among *Phox2b^27ala/+^* mice, *Egr2^cre^* ^/^ *^+^*; *Phox2b^27ala/+^* mice, and their wild‐type (WT) littermates. To test this hypothesis, we examined and compared several complexity metrics across these populations at different developmental stages. We subsequently applied similar analyses to the breathing patterns of human patients with *Phox2b* mutations, adopting an inferential approach to gain phenomenological insights into the potential lesional consequences of these mutations.

## 2 Materials and Methods

### 2.1 Characterization of Ventilatory Activity

#### 2.1.1 Assessment of Breath‐by‐Breath Variability

In all analyzed animal spirograms, ventilatory cycles were identified automatically, and their periods were measured using LabChart (v7.1, ADInstruments, Castle Hill, Australia, RRID:SCR_001620) over the final 3 min of the available 5‐min recordings to maximize the likelihood of stationarity. Variability was assessed by calculating the coefficient of variation (CV), defined as the ratio of the standard deviation to the mean, for the ventilatory period during the selected time frame.

For the human spirograms, we calculated the CV for tidal volume (*V*_T_), ventilatory period (*T*_TOT_), and instantaneous ventilation (*V*′_I_ = *V*_T_/*T*_TOT_) using custom macro commands developed in Matlab (MathWorks Inc., Natick, MA, USA, RRID:SCR_001622).

#### 2.1.2 Assessment of Complexity

In the context of time series analysis, mathematical complexity refers to the degree of nonlinearity and dynamical richness of the underlying system. It captures how unpredictable or irregular a signal is, beyond what can be explained by linear models. In this study, complexity detection was performed using the noise titration method, as previously described (Poon and Barahona 2001; Roulin et al. 2011) and applied in various animal and human models, owing to its robustness in analyzing short and noisy time series (Barahona and Poon 1996; Poon and Barahona 2001). Noise titration begins with nonlinearity detection (Barahona and Poon 1996), which compares the investigated signals to a family of linear and nonlinear polynomial autoregressive models based on Volterra–Wiener–Korenberg series with varying degrees of nonlinearity (*d*) and embedding dimensions (*K*). The linearity hypothesis is tested against the nonlinearity hypothesis using parametric (*F*‐test) and nonparametric (Whitney–Mann) statistical methods. Rejection of the null hypothesis (linearity) indicates that the signal dynamics are nonlinear. The amount of white noise required to make the linearity hypothesis acceptable defines the noise limit (NL), which serves as an estimate of the signal's complexity. Of note, verification of the null hypothesis indicates that the series is not complex or that the complexity that it contained has already been neutralized by the background noise (NL = 0) (Wysocki et al. 2006). In this study, calculations were performed with *d* = 4 and Kappa = 6, in accordance with Roulin et al. (2011). The noise titration process was repeated five times for each spirogram, and the final NL value was the average of these iterations. All calculations were performed on a computing cluster at Sorbonne Université Paris, using an Octave routine (GNU Octave, version 2.1.73, RRID:SCR_014398).

#### 2.1.3 Assessment of Sensitivity to Initial Conditions

Sensitivity to initial conditions was assessed using the largest Lyapunov exponent (LLE), which quantifies the average rate of exponential divergence of nearby trajectories in various directions within phase‐space. For signals with a nonzero NL, the spectrum of Lyapunov exponents was calculated using a polynomial interpolation approach (Briggs 1990) implemented with Dataplore (Datan, Teltow, Germany, RRID: not available). The parameters were set as follows: The number of neighbors was fixed at 28, the polynomial degree was set to 2, and the folding dimension was determined individually for each time series (Briggs 1990).

### 2.2 Animal Study

The analyses were conducted on signals obtained from the *Phox2b^27Aala/+^* and *Egr2^cre^* ^/^ *^+^*; *Phox2b^27ala/+^* mice populations studied previously (RRID:IMSR_EM:02134 and RRID:IMSR_EM:02146, respectively) (Dubreuil et al. 2008; Ramanantsoa et al. 2011) and their WT counterparts. A summary of the observations from these corresponding studies is provided in Figure 1.

#### 2.2.1 Animal Populations

A total of 141 mice, previously described in two publications (Dubreuil et al. 2008; Ramanantsoa et al. 2011), were included in this study. This cohort comprised 15 *Phox2b^27ala/+^* mice and 43 WT mice, all studied within 20 min of birth—P0 development stage (Dubreuil et al. 2008). Additionally, 40 *Egr2^cre^* ^/^ *^+^*; *Phox2b^27ala/+^* mice were analyzed at various developmental stages (P2, *n* = 10; P9, *n* = 9; P22, *n* = 12; adults, *n* = 9) and compared to WT counterparts at the same stages (P2, *n* = 13; P9, *n* = 13; P22, *n* = 8; adults, *n* = 9).

#### 2.2.2 Ventilatory Signals and Recording Conditions

Spirograms were recorded noninvasively using plethysmography in unanesthetized, intact animals, as previously described in detail (Dubreuil et al. 2008; Ramanantsoa et al. 2011). At P0 (*Phox2b^27ala/+^* mice), tidal volume was recorded for 5 min in room air, followed by 5 min of exposure to a hypercapnic atmosphere (8% CO_2_/21% O_2_/71% N_2_), and then a return to room air for a 10‐min post‐hypercapnia period (postCO_2_). At postnatal day 2 (P2) and day 9 (P9) (*Egr2^cre^* ^/^ *^+^*; *Phox2b^27ala^* ^/^ *^+^* mice), the protocol consisted of 10 min in room air, 5 min in hypercapnia, and another 10 min in room air. For *Egr2^cre^* ^/^ *^+^*; *Phox2b^27ala^* ^/^ *^+^* mice at P22 and in adulthood, the recordings included 5 min in room air, 5 min in hypercapnia, and a subsequent 5 min in room air. All signals were digitized at a sampling rate of 100 Hz and stored in text format for subsequent analysis. Of note, movement artifacts characteristic of wakefulness were identified and removed (Dubreuil et al. 2008; Ramanantsoa et al. 2011), restricting the analysis to behavioral quiescence.

#### 2.2.3 Statistical Analysis

The distribution of variables was assessed for normality using the Shapiro–Wilk test. Gaussian data are summarized as mean ± standard deviation, whereas non‐Gaussian data are summarized as median, interquartile range, and range. The proportion of NL‐positive animals between mutant and WT mice was compared using a Chi‐squared test without Yates correction. The combined effects of the mutation and hypercapnia were analyzed at each developmental stage using either a two‐way ANOVA with one repeated factor followed by Bonferroni post hoc tests (for Gaussian data) or the Scheirer–Ray–Hare test followed by Wilcoxon paired tests (for non‐Gaussian data). Developmental changes in ventilatory variability, complexity, and sensitivity to initial conditions (P0 to adulthood in WT mice and P2 to adulthood in *Egr2^cre^* ^/^ *^+^*; *Phox2b^27ala/+^* mice) were assessed using a one‐way ANOVA followed by Tukey post hoc tests (for Gaussian data) or a Kruskal–Wallis test followed by Dunn's post hoc tests (for non‐Gaussian data). For all statistical analyses, differences were considered significant when the probability (*p*) of a type I error was less than 0.05. The analyses were performed using Prism (GraphPad Software, RRID:SCR_002798) or SPSS (IBM Corporation, RRID:SCR_016479).

### 2.3 Human Study

The study was conducted in accordance with the Declaration of Helsinki and approved by the relevant external ethics committee in compliance with French law (Comité de Protection des Personnes Île‐de‐France VI). All participants provided written informed consent.

#### 2.3.1 Patients and Controls

Seven adult patients (five women, two men; mean age 24.4 ± 3 years) having participated in a previously published research (Tremoureux et al. 2014) were studied. Inclusion criteria were a clinical diagnosis of CCHS and the presence of any type of *Phox2b* mutation. Exclusion criteria included gross hypoxemia or hypercapnia during unassisted room air breathing, the presence of respiratory or neurological comorbidities, and being under legal guardianship. Two of the patients studied were tracheotomized. One patient, who was on oral contraception with desogestrel, had regained a ventilatory response to hypercapnia (Straus et al. 2010). The seven patients were compared to a control group of eight healthy individuals (six women, two men; mean age 26.5 ± 2 years) who were free of notable past or present diseases and naïve to respiratory physiology experiments. Data from the control group have previously been used in a published study (Fiamma, Samara, et al. 2007; Fiamma, Straus, et al. 2007). Of note, human respiratory behavior was studied during wakefulness only.

#### 2.3.2 Ventilatory Signals and Recording Conditions

Human ventilatory activity was measured using a calibrated inductance plethysmography vest (Visuresp, RBI, Meylan, France), which has been previously validated for complexity detection (Fiamma, Samara, et al. 2007). This device provides measurements of changes in lung volumes (*V*_PRI_) and ventilatory flow, calculated as its time derivative (*V*′_PRI_). The vest output was digitized at a sampling rate of 40 Hz and subsequently undersampled to 5 Hz for complexity analysis (as described above) (Wysocki et al. 2006). Complexity analysis was performed on both the flow and volume outputs.

Participants were recorded during quiet, spontaneous breathing in room air while comfortably seated in a lounge chair. Soft background music was played at a low volume to create a relaxed environment. Participants were instructed to keep their eyes open and look ahead without fixing their gaze. No additional instructions were provided. Each recording session lasted 10 min.

#### 2.3.3 Statistical Analysis

The distribution of variables was assessed for normality using the Shapiro–Wilk test. Gaussian data are presented as mean ± standard deviation, whereas non‐Gaussian data are summarized as median, interquartile range, and range. Outlier detection was conducted using Grubbs’ tests, with each identified outlier individually examined for potential technical errors. Outliers attributable to technical issues were excluded from the analysis. Gaussian data were compared using an unpaired *t*‐test, with or without Welch's correction depending on the equality of variances. Non‐Gaussian data were compared using the Mann–Whitney *U*‐test. Statistical significance was set at *p* < 0.05, with adjustments for multiple comparisons applied using the Benjamini–Hochberg correction (Haynes 2013). To evaluate the potential impact of including the patient on desogestrel, a sensitivity analysis was performed by excluding this patient's data.

## 3 Results

### 3.1 Animal Study

#### 3.1.1 Breath‐by‐Breath Variability

**FIGURE 2:**
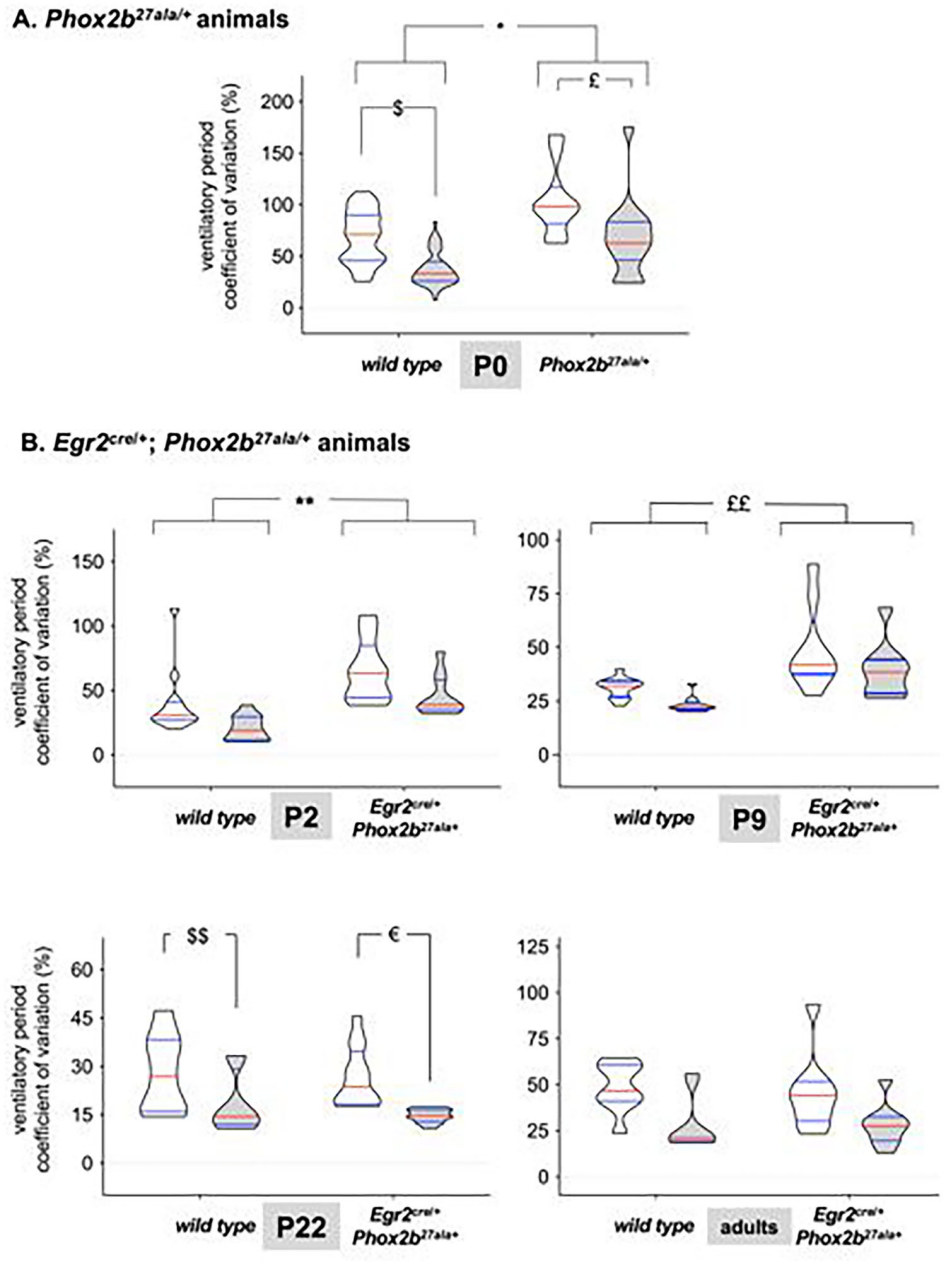
**Breath‐by‐breath variability of the ventilatory period assessed through its coefficient of variation**. (A) Coefficient of variation of the ventilatory period in WT (*n* = 43) and *Phox2b^27ala/+^* mice (*n* = 15) at P0 during room air breathing (white plots) and CO_2_ exposure (gray plots). No differences were detected. (B) Coefficient of variation of the ventilatory period in WT and *Egr2^cre^* ^/^ *^+^*; *Phox2b^27ala/+^* mice at P2 (*n* = 13 vs. *n* = 10, respectively), P9 (*n* = 13 vs. *n* = 9), P22 (*n* = 12 vs. *n* = 8), and adulthood (*n* = 9 vs. *n* = 9) during room air breathing (white plots) and CO_2_ exposure (gray plots). *: *p* = 0.0444, $: *p* < 0.0001£: *p* = 0.0034; **: *p* = 0.025, ££: *p* = 0.026, $$: *p* = 0.0078, € : *p *= 0.0005. Data are presented as truncated violin plots showing the probability density of the distribution, limited to the actual data range to avoid smoothing artifacts, with indication of the median (red horizontal bar) and quartiles (blue horizontal bars).

Across all P0 recordings (*Phox2b^27ala/+^* mice), the CV of the ventilatory period (CV‐TTOT) was higher in *Phox2b^27ala/+^* mice compared to their WT counterparts (*p* = 0.0444). CV‐TTOT decreased with CO_2_ exposure in both *Phox2b^27ala/+^* (*p* = 0.0034) and WT animals (*p* < 0.0001). However, no interaction was observed between genotype and the inhaled gas (*p* = 0.8614; Figure 2A).

In *Egr2^cre^* ^/^ *^+^*; *Phox2b^27ala/+^* mice at P2, CV‐TTOT was higher compared to WT (*p* = 0.025) (Figure 2B). CO_2_ exposure had no effect on CV‐TTOT (*p* = 0.2136), and no genotype‐inhaled gas interaction was observed (*p* = 0.8241). The same pattern was observed at P9 (Figure 2B). At P22, CV‐TTOT did not differ between *Egr2^cre^* ^/^ *^+^*; *Phox2b^27ala/+^* and WT mice. However, CO_2_ exposure decreased CV‐TTOT in both groups (*p* = 0.0078 and *p* = 0.0005, respectively). In adult *Egr2^cre^* ^/^ *^+^*; *Phox2b^27ala/+^* and WT mice, there were no differences in CV‐TTOT during room air breathing or CO_2_ exposure. CO_2_ exposure had no effect on CV‐TTOT in either group (Figure 2A,B). The effects of maturation on respiratory variability were broadly similar in WT and *Egr2^cre^* ^/^ *^+^*; *Phox2b^27ala/+^* mice (Figure S1).

#### 3.1.2 Assessment of Complexity

Across all recordings in this study, 64 out of the 86 (74.41%) from WT mice in room air exhibited a positive NL, compared to 43 out of the 55 recordings (78.18%) from the two mutant lineages (*p* = 0.6104). The proportions of animals with a positive NL under the various experimental conditions are detailed in Table 1. No differences were observed between mutant (both lineages) and WT animals.

**TABLE 1:** Proportions of animals of different age and genotype exhibiting a positive noise limit (NL) in the various experimental conditions (room air breathing or CO_2_ stimulation).

Age	Genotype	Noise limit (NL)
**P0**	W‐T	NL > 0 in all conditions: 21/43 NL > 0 in at least one condition: 17/43 No nonlinearity: 5/43
	*Phox2b^27ala/+^*	NL > 0 in all conditions: 12/15 NL > 0 in at least one condition: 3/15 No nonlinearity: 0/15
**P2**	W‐T	NL > 0 in all conditions: 10/13 NL > 0 in at least one condition: 3/13 No nonlinearity: 0/15
	*Egr2^cre^* ^/^ *^+^*; *Phox2b^27ala/+^*	NL > 0 in all conditions: 9/10 NL > 0 in at least one condition: 1/10 No nonlinearity: 0/10
**P9**	W‐T	NL > 0 in all conditions: 13/13 NL > 0 in at least one condition: 0/13 No nonlinearity: 0/13
	*Egr2^cre^* ^/^ *^+^*; *Phox2b^27ala/+^*	NL > 0 in all conditions: 8/9 NL > 0 in at least one condition: 1/9 No nonlinearity: 0/9
**P22**	W‐T	NL > 0 in all conditions: 7/8 NL > 0 in at least one condition: 0/8 No nonlinearity: 1/8
	*Egr2^cre^* ^/^ *^+^*; *Phox2b^27ala/+^*	NL > 0 in all conditions: 5/12 NL > 0 in at least one condition: 5/12 No nonlinearity: 2/12
**Adults**	W‐T	NL > 0 in all conditions: 9/9 NL > 0 in at least one condition: 0/9 No nonlinearity: 0/9
	*Egr2^cre^* ^/^ *^+^*; *Phox2b^27ala/+^*	NL > 0 in all conditions: 9/9 NL > 0 in at least one condition: 0/9 No nonlinearity: 0/9

**FIGURE 3:**
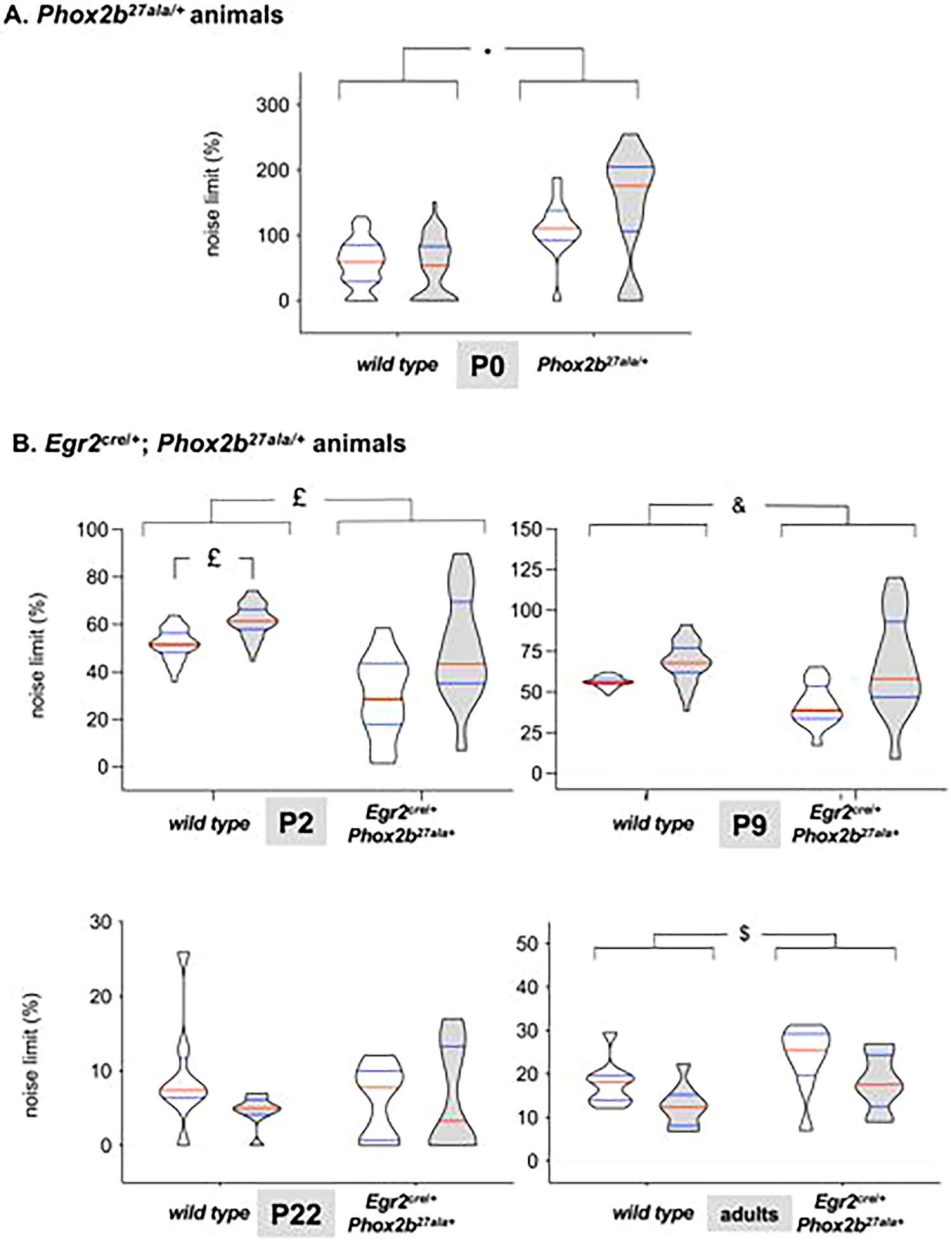
**Breathing complexity assessed through noise titration**. (A) Noise limit computed from the spirograms recorded in WT (*n* = 43) and *Phox2b^27ala/+^* mice (*n* = 15) at P0 during room air breathing (white plots) and CO_2_ exposure (gray plots). *: *p* = 0.0052. (B) Noise limit computed from the spirograms recorded in WT and *Egr2^cre^* ^/^ *^+^*; *Phox2b^27ala/+^* mice at P2 (*n* = 13 vs. *n* = 10), P9 (*n* = 13 vs. *n* = 9), P22 (*n* = 8 vs. *n* = 12), and adulthood (*n* = 9 vs. *n* = 9) during room air breathing (white plots) and CO_2_ exposure (gray plots). *: *p* < 0.05, £: *p* = 0.0419, &: *p* = 0.0495, $: *p* = 0.0201. Data are presented as truncated violin plots showing the probability density of the distribution, limited to the actual data range to avoid smoothing artifacts, with indication of the median (red horizontal bar) and quartiles (blue horizontal bars).

NL was higher in *Phox2b^27ala/+^* mice compared to WT (*p* = 0.00527; Figure 3A). CO_2_ exposure had no effect on NL (*p* = 0.9436), and no interaction between CO_2_ exposure and genotype was observed (*p* = 0.7567; Figure 3A).

During room air breathing, NL was generally higher in *Egr2^cre^* ^/^ *^+^*; *Phox2b^27ala/+^* mice compared to their WT counterparts, with differences observed at P2 (*p* = 0.0419), P9 (*p* = 0.0495), and in adults (*p* = 0.0201; Figure 3B). The effects of CO_2_ exposure on NL were inconsistent across age and genotype (Figure 3B), and no interaction between age and genotype was detected. The effects of maturation on complexity were broadly similar in WT and *Egr2^cre^* ^/^ *^+^*; *Phox2b^27ala/+^* mice (Figure S2).

#### 3.1.3 Assessment of Sensitivity to Initial Conditions

**FIGURE 4:**
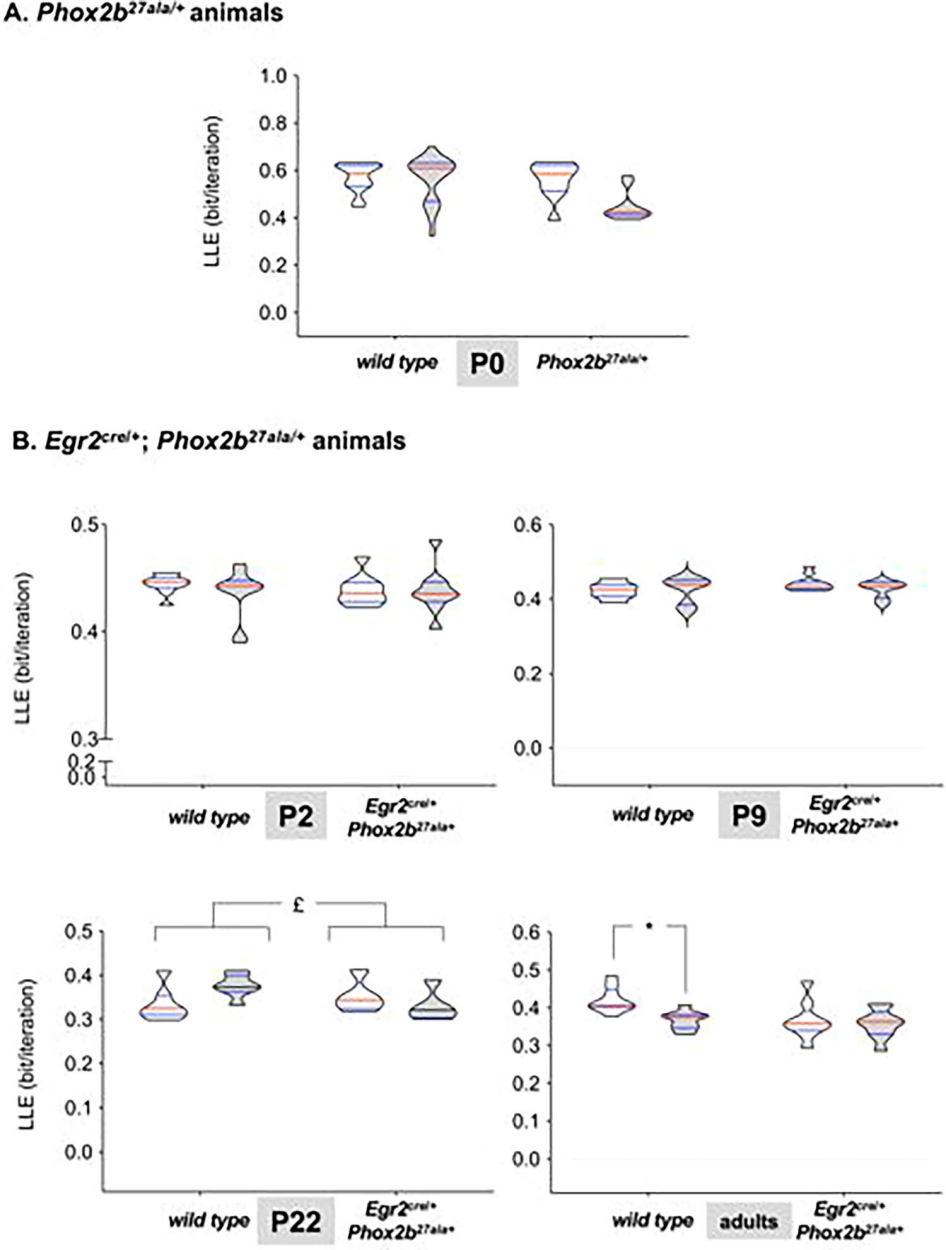
**Breathing complexity assessed through largest Lyapunov exponent**. (A) Largest Lyapunov exponent (LLE) computed from the spirograms recorded in those of the animals where nonlinearity was detected using the noise titration technique (21 WT mice and 10 *Phox2b^27ala/+^* mice) at P0 during room air breathing (white plots) and CO_2_ exposure (gray plots). No differences were detected. (B) Largest Lyapunov exponent computed from the spirograms recorded during room air breathing (white plots) or CO_2_ exposure (grey plots) in those of the animals where nonlinearity was detected using the noise titration technique, at P2 (WT, *n* = 10; *Egr2^cre^* ^/^ *^+^*; *Phox2b^27ala/+^*, *n* = 9), at P9 (*n* = 13 and *n* = 8), at P22 (*n* = 5 and *n* = 7) and in adults (*n* = 9 and *n* = 9). £: *p* = 0.0425; *: *p *< 0.001. Data are presented as truncated violin plots showing the probability density of the distribution, limited to the actual data range to avoid smoothing artifacts, with indication of the median (red horizontal bar) and quartiles (blue horizontal bars).

No difference in LLE was observed between WT and *Phox2b^27ala/+^* mice (*p* = 0.1844). CO_2_ exposure did not affect LLE values (*p* = 0.3269), and no interaction between genotype and breathing condition (room air vs. CO_2_) was detected (*p* = 0.2366; Figure 4A). No differences in LLE were observed between WT and *Egr2^cre^* ^/^ *^+^*; *Phox2b^27ala/+^* mice during room air breathing at P2 and P9. However, differences were detected at P22 and in adults (Figure 4B). CO_2_ exposure had a significant effect on LLE only in adult mice (Figure 4B). The effects of maturation on complexity were broadly similar between WT and *Egr2^cre^* ^/^ *^+^*; *Phox2b^27ala/+^* mice (Figure S3)

### 3.2 Human Study

#### 3.2.1 Breathing Pattern and Breath‐by‐Breath Variability

Data on breathing patterns and breath‐by‐breath variability are presented in Table 2. No differences were observed between controls and CCHS patients, regardless of whether the patient on desogestrel was included in the analysis.

**TABLE 2:** Breathing pattern variables and breath–breath‐variability in patients with CCHS and controls.

		Patients			
	Controls (*n* = 8)	All (*n* = 7)	Desogestrel excluded (*n* = 6)	*p*	p*
Instantaneous ventilation *V*′_I_ (L/min)	6.34 ± 2.97	6.63 ± 1.10	6.37 ± 1.27	0.880	0.985
Coefficient of variation (%)	20.32 ± 3.90	26.03 ± 4.26	27.48 ± 4.74	0.615	0.541
Tidal volume *V*_T_ (L)	0.45 ± 0.26	0.39 ± 0.06	0.36 ± 0.06	0.787	0.755
Coefficient of variation (%)	21.16 ± 5.26	29.10 ± 3.01	29.83 ± 3.53	0.509	0.479
Ventilatory period *T*_TOT_ (s)	4.15 ± 0.10	3.60 ± 0.19	3.56 ± 0.22	0.615	0.541
Coefficient of variation (%)	13.3 ± 3.78	14.98 ± 1.63	14.98 ± 1.63	0.764	0.755

*Note: p:* comparisons of all patients with the control group; p*: sensitivity analysis excluding the one patient receiving desogestrel.

Abbreviation: CCHS, congenital central hypoventilation syndrome.

#### 3.2.2 Assessment of Complexity

**FIGURE 5:**
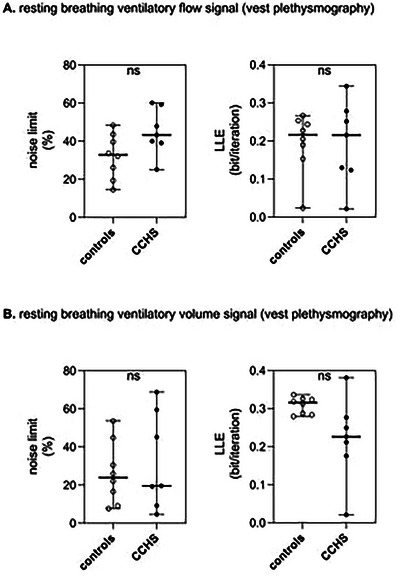
**Noise limit and largest Lyapunov exponent (LLE) in patients with CCHS and controls healthy subjects**. (A) Values calculated from inductance plethysmography vest‐derived ventilatory flow tracings (black circles). (B) Values calculated from inductance plethysmography vest‐derived spirograms (triangles). Horizontal lines represent the mean. NS: no significant difference.

In both the patient and control groups, NL was consistently positive. No between‐group differences were observed when NL was calculated using the inductance plethysmography vest‐derived flow curve, regardless of whether the patient on desogestrel was included or not (*p* = 0.615 and p* = 0.541, respectively). Similarly, no differences were detected when NL was calculated using the inductance plethysmography vest‐derived volume curve (*p* = 0.787 and p* = 0.755, respectively; Figure 5).

#### 3.2.3 Assessment of Sensitivity to Initial Conditions

The Lyapunov exponent spectrum of all signals satisfied the necessary criteria for chaos qualification: at least one positive exponent, at least one negative exponent, and a negative sum of all exponents. No between‐group differences were observed when LLE was calculated from the inductance plethysmography vest‐derived flow curve, regardless of whether the patient on desogestrel was included (*p* = 0.764 and p* = 0.808, respectively). Similarly, no differences were detected when LLE was calculated from the inductance plethysmography vest‐derived volume curve, with or without desogestrel administration (*p* = 0.880 and p* = 0.936, respectively; Figure 5).

## 4 Discussion

### 4.1 Summary of Results

This study demonstrates that mice lacking a functional RTN/pFRG due to a *Phox2b* mutation exhibit greater breath‐by‐breath variability compared to their WT counterparts during early developmental stages, a difference that diminishes with maturation. The mice from both mutant lineages consistently display increased ventilatory complexity, as assessed using the noise titration method, with a higher degree of complexity than WT individuals. Although CO_2_ exposure generally reduces breath‐by‐breath variability, it does not influence ventilatory complexity. Additionally, adult humans carrying a *Phox2b* mutation, which may putatively prevent the development of a functional RTN/pFRG, show no differences in breath‐by‐breath variability or ventilatory complexity compared to controls. Collectively, these findings suggest that the RTN/pFRG is not necessary for mammalian breathing to exhibit mathematical complexity during resting breathing, similar to how the gill oscillator is not necessary for amphibian breathing to demonstrate mathematical complexity. Of notice, these results do not exclude a contribution of the RTN/pFRG to ventilatory complexity when this structure contributes to increased breathing rate during exercise (Hérent et al. 2023) or modified breathing patterns like sighing (Li et al. 2016).

### 4.2 Comparision With Literature Data

Our data are the first to describe breath‐by‐breath variability and ventilatory complexity in *Phox2b* mutant mice. We found few mouse data relevant for comparison with our control data, none of which pertained to ventilatory complexity. Han et al. (2001) reported data allowing to approximate breathing frequency coefficients of variation to about 8% in C57BL/6J unanesthetized, unrestrained, room‐air‐breathing adult mice and about 13% in A/J mice. Lee et al. (2011) reported data allowing to approximate breathing period coefficients of variation to about 5% in B6/129 adult mice. The same figure can be calculated from data by Nielsen et al. (1993) in restricted CF‐1 adult mice. The coefficients of variation observed in our WT mice are well above these values (Figure 2). We have no firm explanation for this discrepancy with literature data. It could stem from several factors, including the genetic strain used or differences in measurement methods (e.g., invasive vs. plethysmography), but also differences in developmental stages. Breathing variability indeed decreases with maturation during the neonatal period, in a manner that parallels the progressive decrease in active sleep (Durand et al. 2005). Importantly, breath‐to‐breath variability decreased under hypercapnic conditions, in a manner similar to what has been described with hypoxic stimulation (Han et al. 2001). Of note, hypoxia or hypercapnia secondary to the effect of the mutation on breathing control could, in principle, confound the interpretation of ventilatory complexity in mutant mice. This is, however, unlikely to be the case, because in conditional mutants at P9 arterial PCO_2_ was within the normal range and not different from controls (40.0 ± 4.5 mmHg in mutants vs. 39.8 ± 3.5 mmHg in controls) (Ramanantsoa et al. 2011), indicating that these animals are not chronically hypercapnic at this developmental stage. Moreover, ventilatory complexity was comparable at P2 and P9, suggesting that its preservation reflects the effect of the mutation itself rather than consequences of altered blood gases.

Regarding the human data, the NL values observed in our healthy control participants align with those previously reported in other studies (Samara et al. 2009; Straus et al. 2021; Wysocki et al. 2006), ranging between 10% and 50% for seated ventilatory flow. Similarly, the LLE values observed in our study (between 0.15 and 0.30 bits/iteration) are consistent with previously published data (Wysocki et al. 2006).

### 4.3 Physiological Considerations

Observing ventilatory complexity in animals lacking RTN/pFRG neurons strongly suggests that the pontomedullary rhythm and pattern generators (including the preBötC) are a highly plausible source of this phenomenon. This interpretation aligns with data demonstrating that these pattern generators can generate and modulate multiple breathing rhythms through intrinsic neuronal properties and dynamic network interactions. Notably, neurons in the preBötC exhibit pacemaker‐like activity and multistability, enabling the production of distinct rhythms under varying external conditions (Lieske et al. 2000; Lü et al. 2019). Moreover, the rhythmogenesis of the preBötC appears quantal, consisting of discrete bursts of activity that are likely to contribute to the complexity of respiratory outputs (Mellen et al. 2003). Its adaptability to perturbations (Marchenko et al. 2016) further supports its critical role in generating complex respiratory patterns. The variability of breathing is increased in mice with functional inactivation of GABA and glycinergic cotransmission in the ventrolateral medulla containting preBötC (Hirrlinger et al. 2019). These observations collectively underscore the preBötC's central role in producing the complex, nonlinear respiratory dynamics we observed. Alternatively, it may result from interactions between the preBötC and other pontomedullary neuron groups involved in respiratory rhythm and pattern production, such as PiCO neurons (Anderson and Ramirez 2017) or Kölliker–Fuse neurons in the pons (John et al. 2024), a hypothesis that would require specific experiments for further elucidation.

At this point, it is worth revisiting the hypothesis of homology between amphibian and mammalian central pattern generators (Vasilakos et al. 2005; Wilson et al. 2006). The ability of the pontomedullary rhythm and pattern generators to generate ventilatory complexity without notable input from the RTN/pFRG is strikingly reminiscent of the amphibian lung rhythm generator's capacity to produce ventilatory complexity without substantial contributions from its gill counterpart. Our findings, therefore, align with the view that the amphibian lung rhythm generator and the mammalian preBötC are functional analogues, a prerequisite for the theory of their evolutionary homology that remains to be tested with comparative transcriptomic approaches.

Our data reveal developmental changes in variability and complexity. This is consistent with established evidence that the role of the RTN/pFRG evolves with maturation—from a structure involved in both rhythm generation and chemosensitivity at birth, to one primarily engaged in central chemoreception, with rhythmogenic activity becoming conditional in adulthood  (Guyenet et al. 2019; Huckstepp et al. 2015; Onimaru et al. 2009).

### 4.4 Study Limitations

Several limitations should be considered when interpreting our findings. First, the genetically modified mice used in this study, particularly the *Egr2^cre^* ^/^ *^+^*; *Phox2b^27ala/+^* model, represent imperfect models of CCHS in humans. Although these mice display a developmental defect in the RTN/pFRG (massive depletion of *Phox2b* cells but with residual 15%–20%), they exhibit partial recovery of chemosensitivity and normalization of ventilation with maturation, allowing them to survive into adulthood. Second, in the human study, we cannot conclusively confirm that the observed patients exhibit a defective RTN/pFRG. The inference regarding the involvement of this structure is indirect, based on the known genetic mutation and associated phenotype. Consequently, the extrapolation of our animal findings to humans should be approached with caution. Third, potential differences in environmental and experimental conditions could influence the variability and complexity metrics. For example, in mice, factors such as the genetic background, age at measurement, and plethysmography protocols may affect the observed outcomes. In humans, although we used validated methods for complexity detection, variations in participant states (e.g., level of relaxation) and measurement duration might have influenced the results. Finally, our statistical power was limited by the relatively small sample size in both the animal and human studies. Although statistically significant differences were detected, future studies with larger cohorts are warranted to confirm our findings and provide more robust conclusions.

## 5 Conclusion

Despite its limitations, this study provides important and novel insights into the origins of ventilatory complexity in mammals. By characterizing breath‐by‐breath variability and ventilatory complexity in *Phox2b* mutant mice and in human patients with CCHS, we offer a new perspective on how respiratory patterns are generated and maintained in the absence of a functional RTN/pFRG. Our results demonstrate that ventilatory complexity persists in mammals even without a functional RTN/pFRG, pointing at the pontomedullary rhythm and pattern generators including the preBötC as its primary source. Moreover, the comparison with amphibian central pattern generators fuels the hypothesis of evolutionary homology between the mammalian preBötC and the amphibian lung oscillator. In addition, our findings in humans highlight the potential for brainstem plasticity or compensation in maintaining complex respiratory patterns despite genetic mutations affecting key respiratory control centers.

## Author Contributions

**Christian Straus**: conceptualization, methodology, validation, supervision, administration, writing (original draft). **Anja Ranohavimparany**: methodology, investigation, data curation, formal analysis, writing (original draft). **Nelina Ramanantsoa**: investigation, formal analysis, writing (review and editing). **Lysandre Tremoureux**: investigation, formal analysis, writing (review and editing). **Maxime Patout**: formal analysis, writing (review and editing). **Marie‐Noëlle Fiamma**: methodology, formal analysis, writing (review and editing). **Florence Cayetanot**: formal analysis, writing (review and editing). **Boris Matrot**: conceptualization, formal analysis, supervision, writing review and editing. **Jorge Gallego**: conceptualization, formal analysis, supervision, writing review and editing. **Laurence Bodineau**: conceptualization, formal analysis, writing (original draft). **Thomas Similowski**: conceptualization, methodology, supervision, administration, writing (original draft): All authors: final approval of manuscript.

## Funding

Anja Ranohavimparany was supported by a “*contrat doctoral*” from the French Ministry of Research. The nonprofit research association “*Association pour le Développement et l'Organisation de la Recherche en Pneumologie et sur le Sommeil*” (ADOREPS), Paris, France contributed to the study funding and served as its legal sponsor.

## Ethics Statement

Animal study: Not applicable (analysis of previously published data). Human Study: The study was conducted in accordance with the Declaration of Helsinki and approved by the relevant external ethics committee in compliance with French law (Comité de Protection des Personnes Île‐de‐France VI).

## Consent

All participants provided written informed consent.

## Conflicts of Interest

The authors declare no conflicts of interest.

## Permission to Reproduce Material From Other Sources

Permissions to reproduce the material used to construct Figure 1 of the manuscript have been obtained from the relevant publishers (see details in the legend to Figure 1).

## Supporting information




**Figure S1**. Effects of maturation on the breath‐by‐breath variability of the ventilatory period assessed through its coefficient of variation.
**Figure S2**. Effects of maturation on ventilatory complexity assessed through the noise titration technique.
**Figure S3**. Effects of maturation on the sensitivity to initial conditions assessed through the largest Lyapunov exponent (LLE).

## Data Availability

The data used in this manuscript will be made available to any researcher upont reasonable request made to Pr Straus (christian.straus@aphp.fr) or Pr Similowski (thomas.similowski@sorbonne-universite.fr).
