# Supplementary material for: Ventilatory Complexity Persists in Phox2b Mutant Mice Lacking the Retrotrapezoid Nucleus/Parafacial Respiratory Group (RTN/pFRG) and in Humans With Congenital Central Hypoventilation Syndrome
Source: J Comp Neurol. 2025 Dec 3;533(12):e70117. doi: 10.1002/cne.70117 (PMC12673958; doi:10.1002/cne.70117)
Supplement: Supplementary file 1 — Figure S1. Effects of maturation on the breath‐by‐breath variability of the ventilatory period assessed through its coefficient of variation. Figure S2. Effects of maturation on ventilatory complexity assessed through the noise titration technique. Figure S3. Effects of maturation on the sensitivity to initial conditions assessed through the largest Lyapunov exponent (LLE). [file CNE-533-e70117-s001.docx]

# **Electronic supplement to** *Ventilatory complexity persists in Phox2b mutant mice lacking the retrotrapezoid nucleus/parafacial respiratory group (RTN/pFRG) and in humans with congenital central hypoventilation syndrome* **by** *Christian Straus, Anja Ranohavimparany, et al.*

**Figure S1.** Effects of maturation on the breath-by-breath variability of the ventilatory period assessed through its coefficient of variation during room air breathing, in WT animals (left; P0:n=43; P2,n=13; P9, n=13; P22, n=8; adults: n=9; *: p<0.001) and *Egr2^cre^*^/+^; *Phox2b^27ala/+^* mutants (right; P2, n=10; P9,n=9; P22, n=12; adults: n=9; &: p<0.001; $: p<0.05). Data are presented as truncated violin plots showing the probability density of the distribution, limited to the actual data range to avoid smoothing artefacts, with indication of the median (red horizontal bar) and quartiles (blue horizontal bars).

**
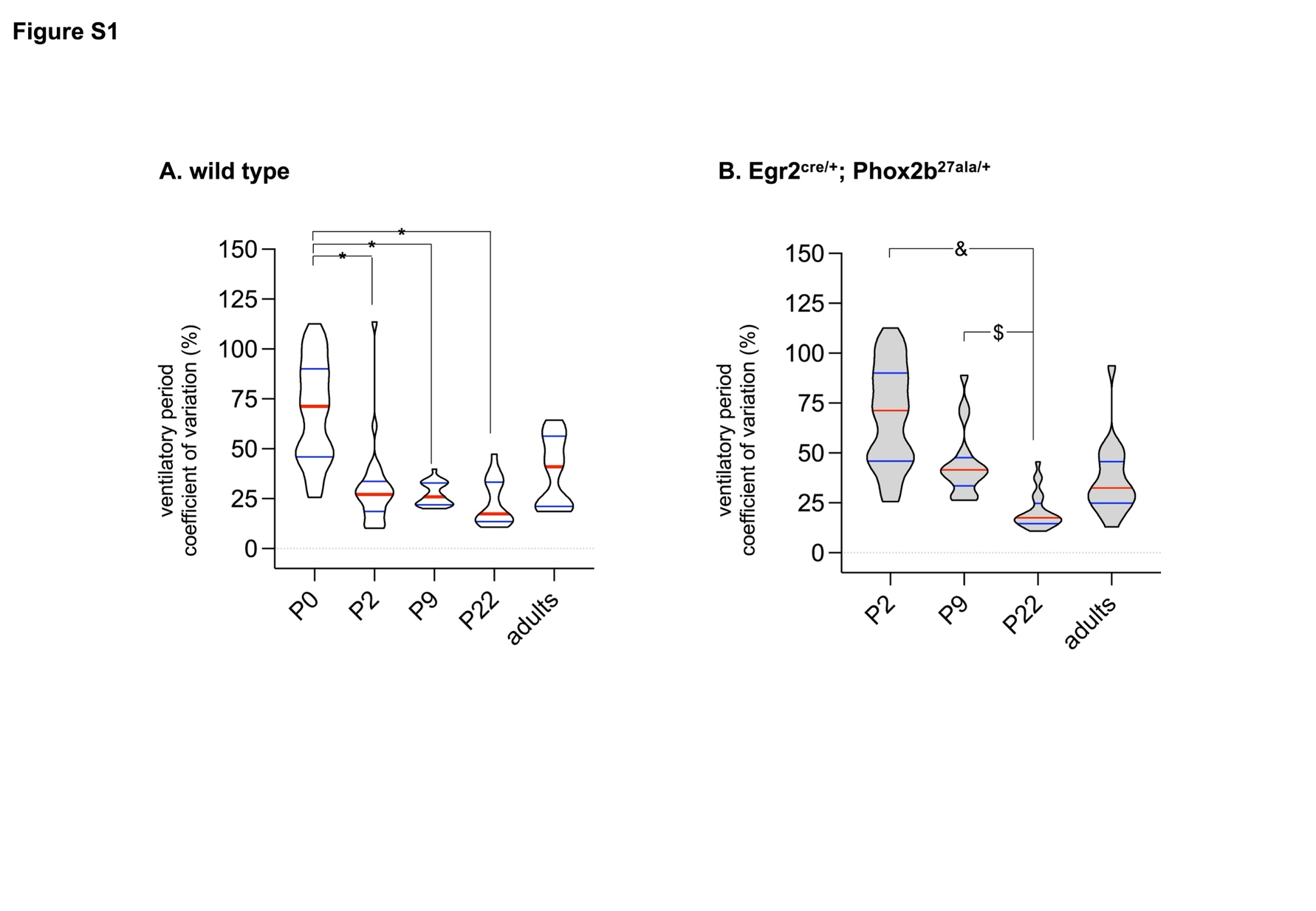
**

**Figure S2.** Effects of maturation on ventilatory complexity assessed through the noise titration technique, in WT animals (left; P0:n=43; P2,n=13; P9, n=13; P22, n=8; adults: n=9; £: p<0.001; $: p<0.05; €: p<0.01) and *Egr2^cre^*^/+^; *Phox2b^27ala/+^* mutants (right; P2, n=10; P9,n=9; P22, n=12; adults: n=9; £: p<0.01, &: p<0.001). Data are presented as truncated violin plots showing the probability density of the distribution, limited to the actual data range to avoid smoothing artefacts, with indication of the median (red horizontal bar) and quartiles (blue horizontal bars).

**
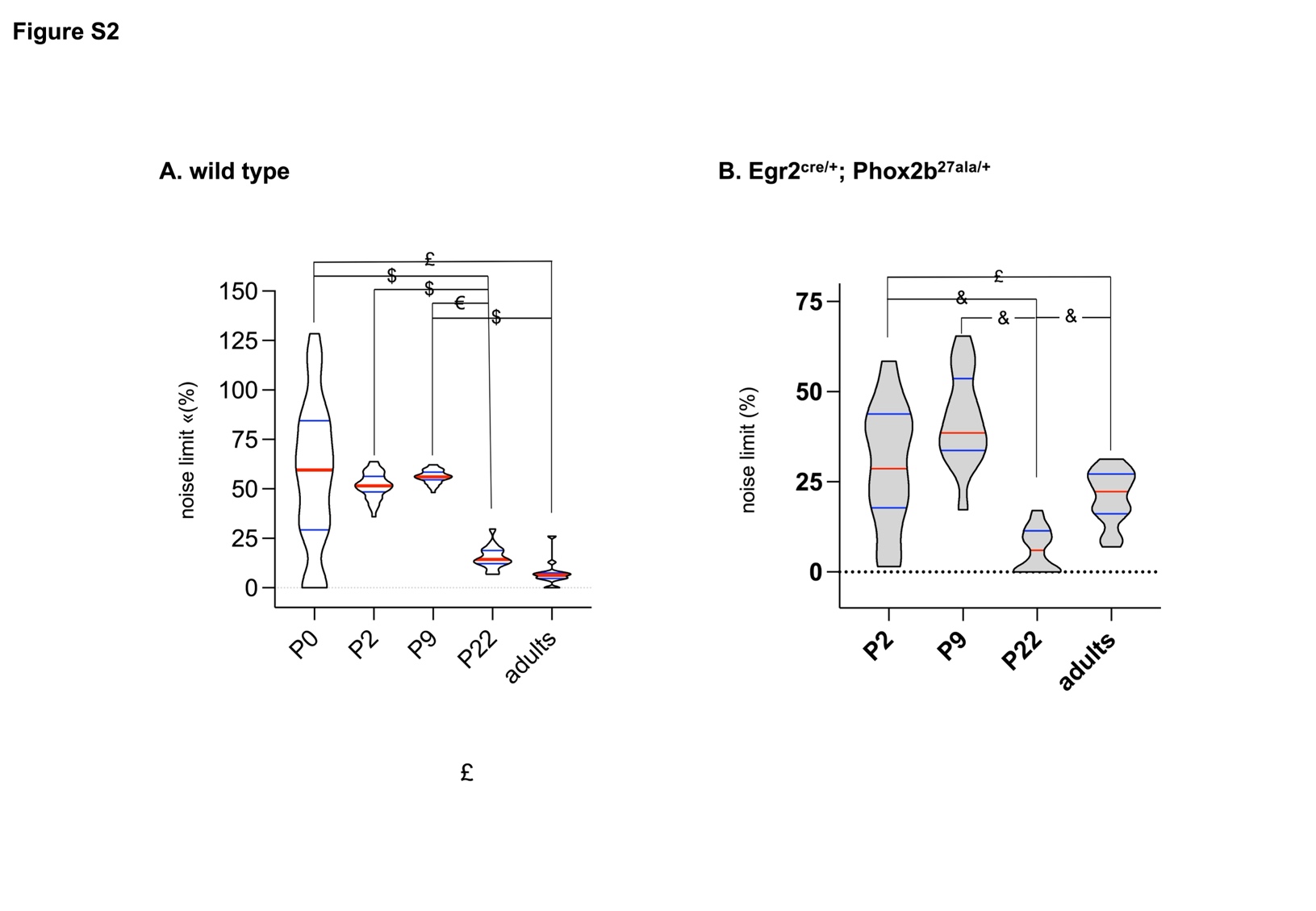
**

**Figure S3.** Effects of maturation on the sensitivity to initial conditions assessed through the largest Lyapunov exponent (LLE) in WT animals exhibiting ventilatory complexity (left; P0: n=21; P2, n=10; P9, n=13; P22 ,n=7; adults: n=9; £: p < 0.001; $: p<0.05) and *Egr2^cre^*^/+^; *Phox2b^27ala/+^* mutants exhibiting ventilatory complexity (right; P2:n=9; P9: n=8; P22:n=5; adults, n=9; £: p<0.05; $: p<0.01). Data are presented as truncated violin plots showing the probability density of the distribution, limited to the actual data range to avoid smoothing artefacts, with indication of the median (red horizontal bar) and quartiles (blue horizontal bars).

**
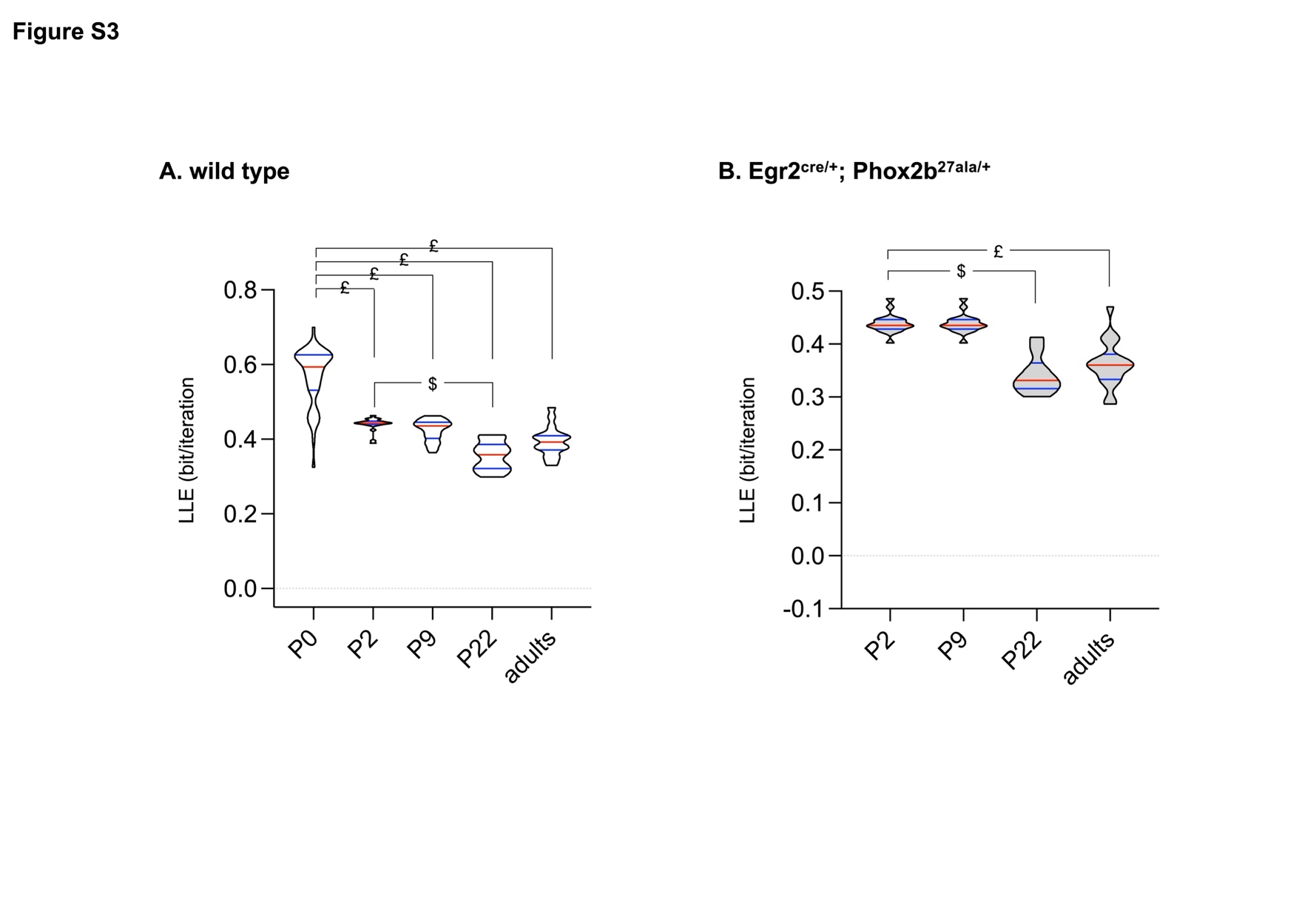
**
